# A Unified Gas–Liquid Carbonation Platform for Habit-Controlled Calcite Nanostructures

**DOI:** 10.3390/nano16140851

**Published:** 2026-07-10

**Authors:** Seungyeol Lee, Juhwan Woo, Chul Woo Rhee

**Affiliations:** 1 Department of Earth and Environmental Sciences, Chungbuk National University, Cheongju 28644, Republic of Korea; jhonta@chungbuk.ac.kr (J.W.); gloryees@chungbuk.ac.kr (C.W.R.); 2 Nano Calcium Korea Co., Ltd., Chungdae-ro, Seowon-gu, Cheongju 28644, Republic of Korea

**Keywords:** calcium carbonate, calcite, morphology control, CO_2_ mineralization, whisker, spindle, nanoparticles

## Abstract

Calcite habit engineering offers a route to transform CO_2_ mineralization from bulk sequestration into value-added nanomaterial production. Here, we demonstrate that additive chemistry and seeding strategy can serve as separable, recipe-level levers for directing calcite habit formation within a unified CaO/Ca(OH)_2_ gas–liquid carbonation platform. This strategy highlights how solution-mediated habit control can bridge fundamental calcite crystallization mechanisms with scalable CO_2_ utilization and value-added carbonate nanomaterial production. Sodium glutamate yielded ~100 nm rhombohedral nanoparticles, staged MgSO_4_/ZnSO_4_ dosing produced whisker-like crystalline nanorods with aspect ratios of 4–7, and two-step seeded carbonation with NH_4_Cl generated fusiform spindle subunits that assembled into hierarchical rosette architectures. X-ray diffraction confirmed calcite as the only crystalline calcium carbonate phase detected under the present measurement conditions, with no detectable aragonite or vaterite reflections. SEM/TEM revealed distinct primary-subunit architectures, including internal striations in spindle particles indicative of oriented attachment. Thermogravimetry, N_2_ physisorption, and EDS further distinguished the products and showed that Mg/Zn/S modifiers in the whisker route are retained predominantly at crystal surfaces rather than incorporated into the calcite lattice. These results define calcite habit control through two independent levers: additive-driven facet selectivity and kinetic decoupling of nucleation from growth/assembly. The platform links scalable synthesis, CO_2_ utilization, and functional carbonate design.

## 1. Introduction

Calcite (CaCO_3_), the thermodynamically stable polymorph of calcium carbonate, is a high-volume material across the pharmaceutical, paper, polymer, paint, and construction industries [1,2], and is simultaneously the most stable terminal sink for CO_2_ captured by reaction with calcium-bearing alkaline precursors through the industrially mature slaking–carbonation sequence (CaO → Ca(OH)_2_ → CaCO_3_) [3,4,5,6]. Its commercial value is governed not only by chemical purity but also by particle habit, size distribution, surface accessibility, and compatibility with target applications: ground calcium carbonate inherits the habit of the parent rock and serves only as a low-value bulk filler, whereas precipitated calcium carbonate (PCC), built de novo, allows size, habit, polymorph, and cation doping to be tuned through supersaturation, temperature, and additive chemistry [7,8]—a designability reflected in a substantial price premium relative to bulk ground calcium carbonate, reflecting the higher value of morphology-controlled precipitated calcium carbonate [1,2,5,9].

Within PCC, calcite habit is governed by the dominance of the {104} cleavage surface, the lowest-energy termination of the *R*$\bar{3}$*c* framework: under unperturbed growth, the six equivalent {104} faces advance at comparable rates, and the crystal relaxes into a near-equant rhombohedron [10,11,12]. Departing from this default requires molecular-scale kinetic intervention—facet-selective step pinning by site-specific adsorbates [13,14,15], or non-classical pathways in which clusters, amorphous precursors, or pre-formed crystallites act as growth units [16,17,18]. Both are well documented, yet their scalable translation remains limited: the most accessible 1D CaCO_3_ habits are metastable aragonite needles [8,19,20], and hierarchical superstructures are typically produced by polymer-templated, membrane-confined, or biomimetic routes that resist scale-up [21,22,23]. However, scalable gas–liquid carbonation routes capable of delivering multiple calcite habits through controlled solution chemistry remain rarely demonstrated.

To address this challenge, we pursue a single specific aim: to establish that calcite habit on a fixed gas–liquid carbonation platform is governed by two separable, recipe-level control levers—additive-directed facet selectivity and seeding-controlled separation of nucleation from growth. The three morphologies reported here are not independent targets but the minimal set of demonstrations required to validate this two-lever framework, since a framework claim of this kind can only be established by showing that distinct habits emerge from an otherwise identical process when the additive/seeding recipe alone is varied. On a single CaO/Ca(OH)_2_ gas–liquid carbonation platform operated at the 20 kg slurry scale within a fixed envelope (10–30 °C; CO_2_ flow 50–150 L·min^−1^·kg^−1^ Ca(OH)_2_; pH endpoint 6.8–7.0), three crystallographically equivalent but morphologically distinct calcite products are accessed by varying additive composition/timing and seed-mediated growth strategy while keeping the reactor configuration, slaking chemistry, gas–liquid contacting mode, and pH endpoint constant: equant ~100 nm rhombohedral nanoparticles, whisker-like crystalline nanorods (aspect ratio 4–7), and fusiform spindle subunits (0.1–0.3 × ~0.8 μm) that self-assemble into hierarchical superstructures. Multi-technique characterization yields cross-corroborating fingerprints of each habit: three independent observations converge on surface—rather than bulk—retention of the Mg/Zn/S modifiers in the whisker route (consistent with facet-selective step pinning), and TEM of an isolated spindle particle resolves internal striations, providing direct microstructural evidence of oriented attachment. Together these results define a predictive design framework for calcite habit engineering—one in which morphology is parameterized by two independent levers, additive facet selectivity and the kinetic decoupling of nucleation from growth—and position morphology-engineered calcite as a value-added co-product of carbon mineralization.

## 2. Materials and Methods

### 2.1. Materials

Calcium oxide (CaO, ≥98% purity, particle size < 75 µm; Sigma-Aldrich, St. Louis, MO, USA) served as the calcium precursor for all three routes, and high-purity CO_2_ gas (99.5%; Linde Korea, Seongnam, Republic of Korea) was used as received. Deionized water (resistivity > 18.0 MΩ·cm) was used throughout. Magnesium sulfate heptahydrate (MgSO_4_·7H_2_O, ≥99%), zinc sulfate heptahydrate (ZnSO_4_·7H_2_O, ≥99%), ammonium chloride (NH_4_Cl, ≥99%), sodium glutamate monohydrate (≥99%), and sulfuric acid (H_2_SO_4_, 95–98%) were obtained from Sigma-Aldrich (St. Louis, MO, USA) and used as received. All reagents were of analytical or industrial grade as appropriate to the route.

### 2.2. Synthesis of Habit-Controlled Calcites

All three products were synthesized on a gas–liquid carbonation platform at the 20 kg slurry scale [5]. The reactor was a stainless-steel cylindrical vessel fitted with an overhead mechanical agitator and a bottom-mounted CO_2_ gas ejector designed to enhance gas–liquid mass transfer and ensure uniform CO_2_ dispersion throughout the slurry. Slaking of CaO was carried out in a separate hydration tank, where the exothermic reaction CaO + H_2_O → Ca(OH)_2_ produced a milk-of-lime slurry at pH 11–12. This conditioned slurry was then transferred to the carbonation reactor for the gas–liquid reaction Ca(OH)_2_ + CO_2_ → CaCO_3_ + H_2_O. All routes used the same CaO/Ca(OH)_2_ gas–liquid carbonation platform and comparable low-temperature, near-neutral endpoint conditions, whereas the gas composition, flow rate, additive timing, and seeding protocol were adjusted according to the target morphology. The pH and slurry temperature were monitored continuously throughout each batch to track reaction progress; no active temperature control was applied at any point during the carbonation step.

Route I employed a single-stage carbonation with a mild organic modifier introduced before CO_2_ injection. Sodium glutamate (0.1–2.0 phr; parts per hundred parts Ca(OH)_2_ by mass) was dissolved in the conditioned Ca(OH)_2_ slurry under continuous agitation at ambient temperature. CO_2_ was then introduced through the bottom-mounted ejector at 50–150 L·min^−1^·kg^−1^ Ca(OH)_2_, and the reaction was carried to a pH endpoint of approximately 7.0. The product slurry was either retained as an aqueous dispersion or dewatered by filtration and dried—preferably by microwave-assisted drying to preserve particle morphology—to give a free-flowing white rhombohedral calcite powder with an average particle size of ~100 nm [5].

Route II used staged dual-additive dosing designed to drive facet-selective step pinning by Mg^2+^, Zn^2+^, and SO_4^2−^_. Each batch began at a slurry temperature of ~14 °C. Immediately before CO_2_ injection, solid MgSO_4_ was added to the conditioned Ca(OH)_2_ slurry at a molar ratio of 0.01–0.03 relative to Ca(OH)_2_ and stirred long enough to disperse Mg^2+^ and SO_4^2−^_ homogeneously before nucleation. This pre-addition exploits the well-established preferential binding of Mg^2+^ to the acute step edges of calcite {104} faces, suppressing lateral step propagation during the earliest stages of crystallization [10]. Carbonation was then initiated by continuously injecting CO_2_ through the gas ejector at 100 L·min^−1^·kg^−1^ Ca(OH)_2_. Concurrent with CO_2_ injection, solid ZnSO_4_ was introduced at a molar ratio of 0.02–0.15 relative to Ca(OH)_2_—either alone or together with H_2_SO_4_ at a ZnSO_4_:H_2_SO_4_ mass ratio between 10:1 and 1:1—to maintain the active Zn^2+^ surface population during the period of fastest crystal growth. No active cooling was applied, and the bulk slurry temperature rose spontaneously from ~14 °C to ~30 °C over the ~6 h batch through the exothermic carbonation enthalpy (ΔH^°^_rxn_ ≈ −113 kJ mol^−1^). The moderate-temperature carbonation condition was selected to favor calcite formation while limiting kinetic pathways that promote metastable calcium carbonate polymorphs: it lies well below the threshold at which aragonite formation becomes kinetically favored in Mg^2+^-bearing carbonation systems, while still providing adequate driving force for calcite nucleation and limiting Ostwald ripening of the primary nanorods. The reaction was terminated when the slurry pH reached ~6.8, and the product slurry was dewatered and dried at room temperature without external heating. The dried product was then deagglomerated and classified to yield the final whisker powder.

Route III deliberately separated nucleation from habit-selective growth across two distinct stages. In Stage 1 (seed generation), a milk-of-lime [Ca(OH)_2_] slurry was wet-milled with a sand-mill (SM) disperser to achieve a uniform particle-size distribution. The SM lime slurry was adjusted to a solids content of 3–10 wt% at 10–20 °C and then carbonated with a gas mixture of 20–40 vol% CO_2_ (balance N_2_) at a flow rate of 40–100 L·min^−1^·kg^−1^ Ca(OH)_2_. Carbonation was terminated at pH ≈ 6.8, yielding a stable colloidal CaCO_3_ seed suspension with an average particle size of ~0.04 μm. In Stage 2 (hierarchical growth and assembly), a separate SM lime slurry (3–10 wt%, 10–30 °C) was prepared and pre-dosed with NH_4_Cl as a crystal-habit modifier at a molar ratio of 0.01–0.20 relative to Ca(OH)_2_. This NH_4_Cl-modified slurry was introduced into the primary seed suspension at a ratio of 10–30 mol Ca(OH)_2_ per mol of pre-formed seeds. Secondary carbonation was then initiated by injecting 20–40 vol% CO_2_ at 20–80 L·min^−1^·kg^−1^ Ca(OH)_2_, and the NH_4_Cl-buffered ionic environment promoted anisotropic CaCO_3_ growth along the crystallographic *c*-axis on the seed surfaces. The reaction was terminated at pH ≈ 6.8, and the precipitates were recovered by centrifugation to a solids content of approximately 65%.

### 2.3. Characterization

Characterization of the three calcite products was carried out at two facilities at Chungbuk National University (CBNU). Crystallographic phase analysis and morphological and elemental examination (XRD, SEM, EDS) were performed at the Mineralogy and Mineral Resources Laboratory, Department of Earth and Environmental Sciences, while nanoscale, textural, and thermal analyses (TEM, N_2_ physisorption, TG/DTG) were performed at the CBNU Central Instrumentation Facility.

Powder X-ray diffraction patterns were collected on a MiniFlex 600 diffractometer (Rigaku Corporation, Tokyo, Japan) using Cu Kα radiation (λ = 1.5406 Å) at an accelerating voltage of 40 kV and an emission current of 15 mA. Patterns were recorded over 2θ = 5–90° at a step size of 0.01° and a scan rate of 3° min^−1^. Prior to measurement, the instrument was calibrated against a standard silicon reference material to minimize systematic angular deviations. Phases were identified using the ICDD PDF database (calcite reference PDF 05-0586), and volume-averaged crystallite sizes were estimated from the full width at half maximum of the (104) reflection using the Scherrer equation.

Thermogravimetric and derivative thermogravimetric (TG/DTG) curves were recorded on a NETZSCH TG 209 F1 Libra analyzer (NETZSCH-Gerätebau GmbH, Selb, Germany) running Proteus software (version 8.0). Approximately 6–7 mg of dried sample was loaded into an open Al_2_O_3_ crucible (85 μL) and heated from 30 to 1100 °C at 10 °C min^−1^ under flowing nitrogen (250.0 mL min^−1^ for both the purge and protective streams). A method-specific baseline correction was applied to ensure accurate determination of decarbonation kinetics. Residual masses and peak decomposition temperatures were taken from the TG and DTG traces, respectively; the theoretical 44.0 wt% mass loss for stoichiometric decarbonation of CaCO_3_ to CaO and CO_2_ served as a reference for evaluating the carbonate-dominated composition and additional non-carbonate mass-loss contributions.

Nitrogen adsorption–desorption isotherms were collected at 77 K (−195.8 °C) on a Micromeritics ASAP 2425 analyzer (Micromeritics Instrument Corp., Norcross, GA, USA). Approximately 0.15 g of sample was used per measurement with a 10 s equilibration interval. Prior to measurement, samples were outgassed under vacuum to remove physisorbed water and surface contaminants. Specific surface areas were calculated by the BET method [24] over the linear range 0.05 ≤ P/P_0_ ≤ 0.30, with linearity verified by inspection of the BET-transformed plot [25]. Pore-size distributions and pore volumes were derived from the desorption branch using the BJH model [26] together with the Harkins–Jura statistical thickness equation [27] and the Faas correction, and microporous contributions were assessed by the *t*-plot method.

Morphological features and elemental compositions were examined by scanning electron microscopy (SEM) using a JSM-IT510 microscope (JEOL Ltd., Tokyo, Japan) operating at 10–15 kV in high-vacuum mode. The instrument was equipped with an Oxford Instruments (Abingdon, UK) energy-dispersive X-ray spectroscopy (EDS) detector for qualitative and semi-quantitative elemental mapping. Dried powders were mounted on aluminum stubs with carbon adhesive tape. Primary-particle dimensions (diameter or length, width, and aspect ratio) were measured from more than 100 randomly selected particles per sample (N = 120 for Rn, N = 115 for spindle, and N = 130 for whisker) from SEM images using ImageJ (version 1.54g; National Institutes of Health, Bethesda, MD, USA). EDS spectra were acquired at 15 kV with an acquisition time of 60–120 s per spectrum; the presence or absence of the Mg Kα, Zn Lα, and S Kα lines was used as an indicator of additive-derived species in the whisker product.

High-resolution imaging and structural analysis were carried out on a spherical-aberration (Cs)-corrected transmission electron microscope (JEM-ARM200F NEOARM; JEOL Ltd., Tokyo, Japan) operating at 200 kV. Specimens were prepared by ultrasonically dispersing the powder in ethanol for 10 min to minimize agglomeration; a drop of the resulting suspension was deposited onto a carbon-coated lacey copper grid and dried under ambient conditions prior to analysis.

## 3. Results

### 3.1. Synthesis on the Gas–Liquid Carbonation Platform

The three calcite products were synthesized on the platform of Section 2.2 within a single operating envelope: CO_2_ flow of 50–150 L·min^−1^·kg^−1^ Ca(OH)_2_, continuous mechanical agitation, and a pH endpoint of 6.8–7.0. The protocols differed mainly in the additive system and the seeding strategy, with route-specific adjustments in gas composition and flow rate. Route I (rhombohedral nanoparticles, hereafter Rn) used a mild organic modifier (sodium glutamate) introduced before CO_2_ injection; Route II (whisker) used staged dual-additive dosing, with MgSO_4_ added before CO_2_ and ZnSO_4_ concurrent with it; and Route III (spindle) used two-step seeded carbonation with NH_4_Cl introduced in the Stage 2 growth feed.

### 3.2. Crystallographic Phase Identification

**Figure 1:**
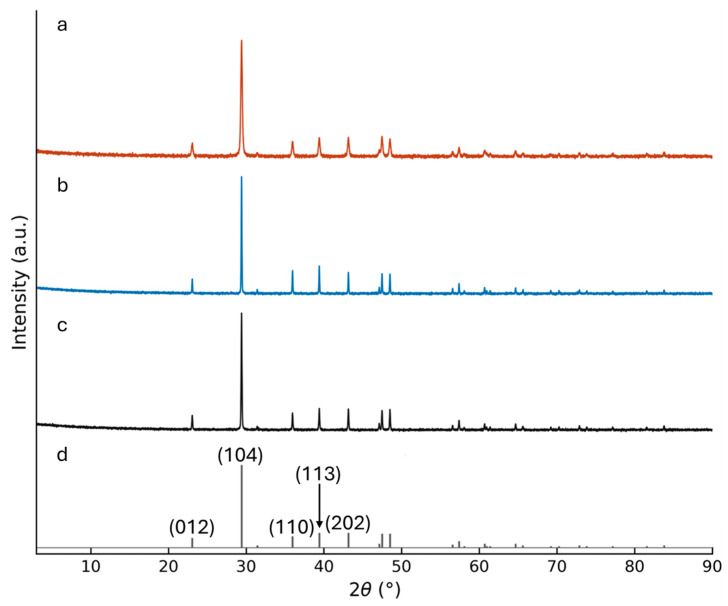
Powder XRD patterns of the three calcite products—(a) rhombohedral ~100 nm (Rn), (b) spindle, (c) whisker, together with (d) the calcite reference pattern (ICDD PDF 05-0586). All reflections index to calcite; no aragonite or vaterite is detected, and no systematic peak shifts occur across the three products.

Powder X-ray diffraction patterns of the three products are shown in Figure 1. In panels a–c, every observed reflection indexes unambiguously to rhombohedral calcite (space group *R*$\bar{3}$*c*) by reference to ICDD PDF 05-0586. The (104) reflection at 2θ ≈ 29.4° dominates each pattern, accompanied by the (012), (110), (113), and (202) reflections at their expected positions. No reflections attributable to aragonite or vaterite were detected under the present measurement conditions, indicating that all three products are dominated by rhombohedral calcite. The three samples are therefore crystallographically equivalent at the bulk-phase level, and the morphological diversity established below arises within a single thermodynamically stable polymorph rather than across different polymorphs.

**Figure 2:**
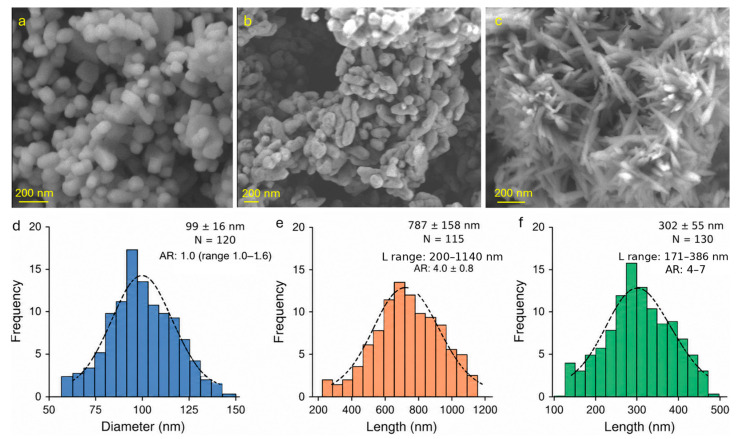
SEM images of the three calcite products at uniform magnification and the corresponding primary-particle size distributions. (**a**) Rn, equant near-rhombohedral particles; (**b**) spindle, fusiform subunits aggregated into hierarchically porous superstructures; (**c**) whisker, rod-like subunits forming a cauliflower-like aggregate (scale bars: 200 nm). (**d**–**f**) Size distributions measured from SEM images.

**Figure 3:**
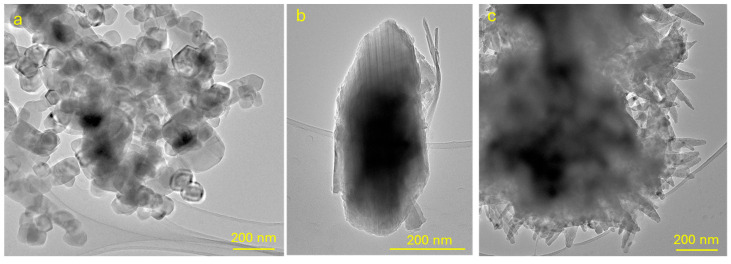
TEM of the three products (200 nm scale bars): (**a**) Rn, discrete {104}-bounded rhombohedra (~50–150 nm); (**b**) an isolated spindle particle (~250–300 × 700–800 nm) with internal contrast striations parallel to the long axis; and (**c**) whisker, rod-like subunits (~30–100 × 100–300 nm) radiating from a dense central core.

Two further crystallographic observations bear directly on the mechanism. First, the (104) reflection of the whisker sample is modestly broader in FWHM (0.163°) than those of the rhombohedral (0.110°) and spindle (0.102°) samples, and the corresponding Scherrer crystallite sizes corroborate the electron microscopy below (Figure 2). The rhombohedral (Rn) domain size (75 ± 8 nm) agrees, within error, with the discrete single-crystal rhombohedra imaged by TEM (~50–150 nm), indicating that each Rn particle is essentially a single coherently diffracting crystal. The spindle domain size (80 ± 9 nm) is far smaller than the overall spindle dimensions (~0.25–0.30 × 0.70–0.80 μm), consistent with each spindle being a composite of smaller, crystallographically co-aligned primary subunits rather than a single crystal—in line with the oriented-attachment striations resolved in Figure 3b. The whisker exhibits the smallest domain size (51 ± 6 nm), comparable to the rod cross-section observed by SEM/TEM (~30–100 nm; Figure 3c); the broadening is therefore dominated by the small primary-subunit size, with any additional microstrain from additive-modified (surface Mg/Zn/SO_4_) growth remaining minor and not separable from the size contribution using a single reflection.

We note that Rietveld refinement and Williamson–Hall analysis were considered as routes to quantitative lattice parameters and microstrain; however, the survey-scan conditions employed here were optimized for phase identification rather than for the counting statistics required by full-pattern refinement, and Williamson–Hall analysis presupposes isotropic size–strain broadening—an assumption poorly satisfied for the strongly anisotropic whisker crystallites, whose direction-dependent domain dimensions would convolute with strain broadening. The absence of systematic peak shifts suggests that any bulk Mg/Zn incorporation, if present, was below the resolution of the present laboratory XRD data; high-resolution synchrotron-based Rietveld refinement capable of resolving lattice parameters and site occupancies below this bound is planned as future work.

### 3.3. Morphology and Primary-Subunit Nanostructure

SEM at uniform magnification (Figure 2) reveals a clear progression in primary-subunit anisotropy, from equant to one-dimensional rod-like. Panel a (Rn) shows equant, near-rhombohedral primary particles of ~50–150 nm aggregated into loosely packed clusters; the slight rounding of the rhombohedral edges at this magnification reflects kinetic moderation of {104} face advancement by the mild organic modifier. Panel b (spindle) shows elongated fusiform primary subunits, ~100–300 nm wide and ~200–1140 nm long (aspect ratio 3–5), aggregated into hierarchically porous, rosette-like superstructures. Panel c (whisker) shows high-aspect-ratio rod-like primary subunits, ~50–100 nm wide and 171–386 nm long (aspect ratio 4–7), randomly oriented into an interlocked, cauliflower-like secondary architecture.

The particle size distributions measured from the SEM images (Figure 2d–f) quantify the uniformity of the three products. The Rn product shows a monomodal, near-Gaussian diameter distribution centered at 99 ± 16 nm (N = 120) with an aspect ratio of 1.0, confirming the equant habit. The spindle subunits show a monomodal length distribution of 787 ± 158 nm (N = 115; range 200–1140 nm) with a mean aspect ratio of 4.0 ± 0.8, and the whisker subunits show a monomodal length distribution of 302 ± 55 nm (N = 130; range 171–386 nm) with aspect ratios of 4–7. The narrow, monomodal character of all three distributions demonstrates that each route delivers a uniform primary-particle population despite the heterogeneous supersaturation environment of the 20 kg reactor, supporting the reproducibility of the recipe-level habit control.

TEM at uniform magnification (Figure 3) resolves the primary-subunit microstructure in greater detail. The Rn product (panel a) consists of discrete, well-faceted rhombohedral primary particles with sharp polyhedral outlines characteristic of {104}-bounded calcite, consistent with well-crystallized rhombohedra formed under near-equilibrium {104} expression. The spindle product (panel b) is shown as an isolated fusiform particle of ~250–300 nm (width) × 700–800 nm (length) that displays pronounced internal contrast striations running parallel to the long axis. Such internal striations are not expected for a simple single-crystal growth history and are more consistent with oriented attachment of smaller anisotropic subunits, indicating that each macroscopic spindle is built from a population of smaller anisotropic primary subunits docked along matching crystallographic faces—a microstructural fingerprint of crystallization by particle attachment [16,17,18]. The whisker product (panel c) reveals a dense, aggregated central core from which discrete 1D primary subunits (~30–100 nm wide, ~100–300 nm long) radiate in random orientations, generating the cauliflower-like secondary architecture seen at the SEM scale. The well-defined, uniform geometry of these radiating subunits confirms that they are discrete crystalline rods rather than continuous fibrous overgrowths.

The three products are thus distinguished not only at the bulk-aggregate level but, more diagnostically, at the level of primary-subunit architecture: discrete equant single crystals (Rn), oriented-attachment composites (spindle), and aggregated 1D crystalline rods (whisker).

### 3.4. Thermal Stability and Surface Chemistry

**Figure 4:**
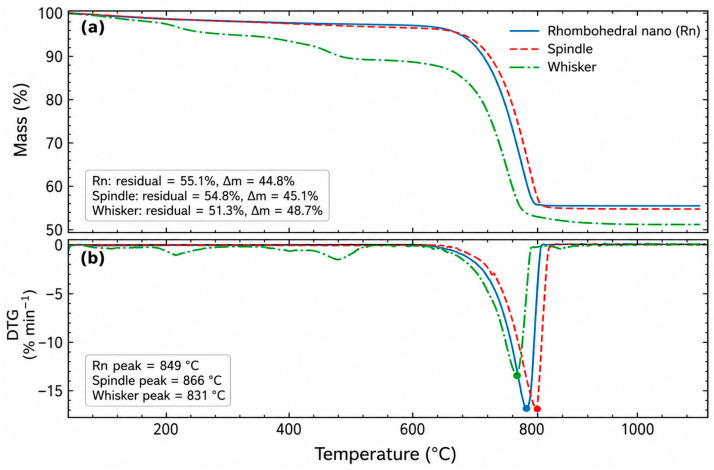
(**a**) TG and (**b**) DTG curves of the three products under flowing N_2_ at 10 °C min^−1^. The whisker profile shows an additional multi-step mass loss (~4.7%) between 200 and 600 °C that is absent in the Rn and spindle profiles; main decarbonation peaks: 849 °C (Rn), 866 °C (spindle), and 831 °C (whisker).

TG and DTG analyses (Figure 4) support the carbonate composition inferred from XRD and reveal morphology-dependent mass-loss behavior that reflects differences in surface chemistry. The TG profiles (Figure 4a) give final residual masses at 1100 °C of 55.1% (Rn), 54.8% (spindle), and 51.3% (whisker), corresponding to total mass losses of 44.9%, 45.2%, and 48.7%. All three losses are close to or exceed the theoretical 44.0 wt% for stoichiometric decarbonation of CaCO_3_ → CaO + CO_2_, consistent with the carbonate identity established by XRD; the excess above 44.0% is examined next.

A morphology-dependent feature appears in the pre-decarbonation region. The Rn and spindle profiles remain essentially flat from ambient temperature to ~650 °C; the small excess losses of 0.9% and 1.2% above the stoichiometric 44.0% can be assigned to desorption of physisorbed water and surface hydroxyls. The whisker profile is qualitatively different, showing a distinct multi-step pre-decarbonation mass loss of roughly 4.7% between 200 and 600 °C. The corresponding whisker DTG trace (Figure 4b) resolves several small, well-separated minima in the 400–600 °C window superimposed on the main decarbonation event—a signature of stepwise decomposition of distinct surface-bound species. The additional mass loss is consistent with dehydration and/or decomposition of additive-derived sulfate, hydroxylated, or surface-associated species, although complementary spectroscopic analysis would be needed for definitive assignment.

The main decarbonation DTG peaks lie at 849 °C (Rn), 866 °C (spindle), and 831 °C (whisker), all within the range characteristic of well-crystallized phase-pure calcite [28,29]. The ordering spindle > Rn > whisker tracks the primary-subunit lateral dimension (spindle ~100–300 nm > Rn ~100 nm > whisker ~50–100 nm), consistent with longer CO_2_ diffusion paths in the larger subunits—an internally consistent thermal fingerprint of the three habits.

### 3.5. Textural Porosity

**Figure 5:**
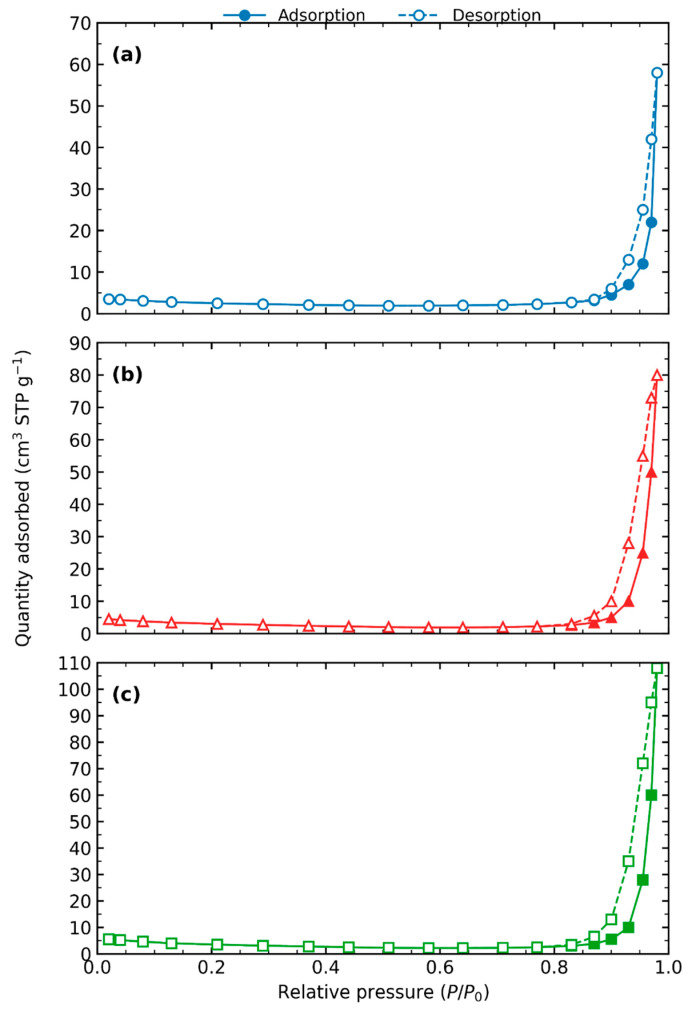
N_2_ adsorption–desorption isotherms at 77 K: (**a**) Rn, (**b**) spindle, (**c**) whisker. Closed and open symbols denote adsorption and desorption branches, respectively. All are IUPAC Type II with H3 hysteresis above P/P_0_ ≈ 0.85. BET areas 17.5 (Rn), 12.4 (spindle), and 9.6 (whisker) m^2^ g^−1^; BJH mesopore modes ~43 nm (Rn), ~53 nm (spindle), and ~49 nm (whisker).

Nitrogen adsorption–desorption isotherms at 77 K (Figure 5) all conform to the IUPAC Type II classification, with H3-type hysteresis loops developing above P/P_0_ ≈ 0.85. Gas uptake is very low across the low- and intermediate-pressure regions, with a sharp uptake only as the saturation pressure is approached—diagnostic of materials whose primary subunits are essentially non-porous, with the only accessible porosity arising from interstitial voids between aggregated subunits.

The maximum quantities adsorbed near P/P_0_ ≈ 0.98 increase as Rn (~58 cm^3^ STP g^−1^) < spindle (~80 cm^3^ STP g^−1^) < whisker (~108 cm^3^ STP g^−1^), and the H3 loop broadens in the same order—both tracking the increasing volume and connectivity of interparticle voids in progressively more anisotropic, more densely interlocked assemblies. BET specific surface areas over the linear range (0.05 ≤ P/P_0_ ≤ 0.30) are 17.5 m^2^ g^−1^ (Rn), 12.4 m^2^ g^−1^ (spindle), and 9.6 m^2^ g^−1^ (whisker), and the BJH desorption-branch mesopore modes lie at ~43 nm (Rn), ~53 nm (spindle), and ~49 nm (whisker), matching the interstitial dimensions of the hierarchical assemblies established by SEM and TEM (Section 3.3) rather than any intraparticle channels.

Two implications follow. First, the textural porosity of the habit-controlled products is set at the level of secondary assembly rather than within the primary crystals—a feature directly traceable to the distinct subunit architectures of the three routes. Second, the moderate surface areas (~10–18 m^2^ g^−1^) are consistent with well-ordered primary subunits and rule out substantial amorphous or microporous contamination. The ordering Rn (17.5) > spindle (12.4) > whisker (9.6) m^2^ g^−1^ is at first sight counterintuitive, since the whisker product comprises the smallest primary subunits (~30–100 nm wide by SEM/TEM). The inversion is resolved by recognizing that the whisker subunits are tightly aggregated around a dense central core (Figure 3c) that occludes much of the geometric subunit surface from N_2_, whereas the loosely aggregated, near-rhombohedral Rn particles (Figure 3a) leave a larger fraction of their surface accessible. The measured BET areas therefore reflect the architecture of the secondary assembly rather than primary-subunit dimensions alone.

### 3.6. Surface Elemental Composition

**Figure 6:**
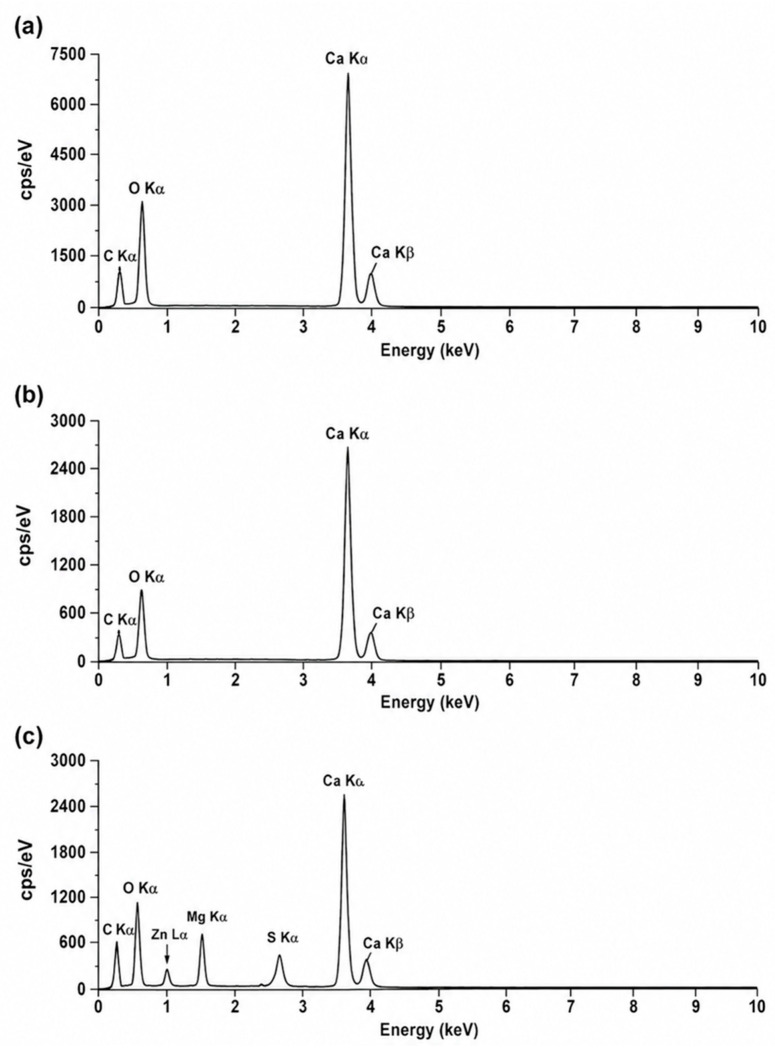
Energy-dispersive X-ray spectroscopy (EDS) of the three habit-controlled calcite products. (**a**) Rn and (**b**) spindle show only C, O, and Ca lines (no Cl); (**c**) whisker shows, in addition, Mg Kα (1.25 keV), Zn Lα (1.01 keV), and S Kα (2.31 keV).

EDS spectra of the three products (Figure 6) provide chemical evidence for the surface-localized retention inferred above from XRD and TGA. The Rn spectrum (panel a) shows only the C Kα, O Kα, Ca Kα, and Ca Kβ lines, with no foreign elements detectable within the EDS limit (~0.1–1.0 wt%). The spindle spectrum (panel b) likewise shows only the C, O, and Ca lines; no Cl signal was detected by EDS, suggesting that residual chloride, if present, was below the EDS detection limit.

The whisker spectrum (panel c) differs. In addition to the C, O, and Ca lines, three diagnostic trace peaks appear—Mg Kα (1.25 keV), Zn Lα (1.01 keV), and S Kα (2.31 keV). Their coexistence and low intensity relative to the Ca Kα line indicate the retention of trace Mg^2+^, Zn^2+^, and SO_4^2−^_ species from the MgSO_4_/ZnSO_4_ additive system that produced the whisker habit.

Three independent lines of evidence collected on the same sample therefore converge on a single conclusion—the Mg/Zn/S species are predominantly surface-associated or retained in near-surface/additive-derived environments rather than extensively incorporated into the bulk calcite lattice: the absence of XRD peak shifts (Section 3.2), the distinctive multi-step pre-decarbonation mass loss between 200 and 600 °C (Section 3.4), and the direct EDS detection of Mg, Zn, and S (this section). This convergence provides a robust empirical foundation for the mechanistic interpretation of the whisker route developed in Section 4.

## 4. Discussion

### 4.1. Crystallographic Framework: The {104} Hegemony and the Kinetic Envelope of Habit Control

Section 3 established that three radically different calcite morphologies—equant rhombohedral nanoparticles, whisker-like crystalline nanorods, and oriented-attachment spindle/rosette assemblies—can be reproducibly accessed on a single CaO/Ca(OH)_2_-based gas–liquid carbonation platform through controlled adjustment of additive chemistry and seeding strategy alone. We interpret this morphological diversity through the lens of {104} step kinetics, the dominant surface-chemical lever available to calcite habit engineers.

Calcite crystallizes in the trigonal system [30], and the {104} cleavage plane represents its lowest-energy surface termination [11,12]. Under unperturbed aqueous growth, the six symmetrically equivalent {104} faces advance at comparable rates, producing the near-equant rhombohedron observed for the Rn product (Figure 2a and Figure 3a) [7,10]. Departures from this default habit require kinetic intervention at the molecular scale, and two broad routes are available: facet-selective suppression of step propagation by site-specific adsorbates or recruitment of non-classical pathways in which clusters, amorphous precursors, or pre-formed crystalline subunits act as growth units rather than individual ions [13,16,17]. The three habit routes reported here populate distinct regions of this conceptual landscape. The Rn route operates close to equilibrium with minimal facet selectivity. The whisker route operates through high facet selectivity within the classical framework. And the spindle route combines low-to-moderate facet selectivity with non-classical assembly. Taken together, the three routes span the kinetic envelope of calcite habit control accessible on the gas–liquid carbonation platform.

A further crystallographic constraint defines the outer boundaries of this envelope. The metastable polymorphs aragonite and vaterite become kinetically competitive at elevated temperatures (above ~50–60 °C) [20,31] or at elevated Mg^2+^/Ca^2+^ activity ratios that stabilize Mg-substituted amorphous calcium carbonate intermediates [19,32,33]. The operating envelope used here—10–30 °C, MgSO_4_ ≤ 0.03 mol/mol Ca(OH)_2_—was deliberately positioned to avoid both regimes. The phase-pure calcite identification across all three products by XRD (Figure 1) confirms that this kinetic boundary management was successful and that the morphological diversity reported here was achieved within a single thermodynamically stable polymorph rather than across different polymorphs.

### 4.2. Facet-Selective Step Pinning in the Whisker Route: Triangulated Evidence for Surface-Localized Mg/Zn/SO_4_ Retention

The mechanistic interpretation of the whisker route is the most consequential of the three discussed here. It bears directly on a long-standing question in calcite habit engineering: whether additive-induced anisotropy arises from bulk lattice substitution of the foreign cations or from their site-specific adsorption at crystal surfaces [10,14,34,35]. Our data consistently favor the surface adsorption hypothesis. Three independent lines of evidence converge on a single consistent conclusion.

First, the powder XRD pattern of the whisker product (Figure 1c) shows no systematic angular shifts in any reflection relative to the rhombohedral and spindle samples or to the calcite reference pattern (ICDD PDF 05-0586). Substantial isomorphous substitution of Mg^2+^ (ionic radius ~0.72 Å) or Zn^2+^ (~0.74 Å) for Ca^2+^ (~1.00 Å) in the calcite framework would generate proportional contractions of the unit-cell parameters, with corresponding angular shifts in the (104), (012), (006), and other reflections [30]. The absence of such shifts within instrumental resolution sets an upper bound on bulk substitution at well below detectable levels.

Second, the TG profile of the whisker product (Figure 4a) shows a distinctive multi-step pre-decarbonation mass loss of ~4.7 wt% between 200 and 600 °C, far exceeding the 0.9–1.2 wt% observed for the rhombohedral and spindle samples in the same temperature range. Several resolved minima appear in the DTG profile within this window (Figure 4b). This thermal signature is consistent with stepwise decomposition of surface-bound species, not with the carbonate-framework decomposition itself, which occurs as a single sharp event at 831 °C in the same profile. The multi-stage character of the pre-decarbonation loss rules out surface dehydration alone (which would produce a single broad event below 300 °C) and points instead to discrete decomposition of multiple surface-retained additive-derived species. This pattern matches what is reported in the literature for Mg- and Zn-sulfate surface complexes on carbonate minerals [15,28].

Third, EDS analysis (Figure 6c) directly identifies the chemical species responsible. The Mg Kα, Zn Lα, and S Kα characteristic lines appear in the whisker product at trace intensities relative to the Ca Kα line. These same lines are absent in the rhombohedral and spindle products (Figure 6a,b), tying the surface-retained species explicitly to the additive system used in the whisker synthesis (MgSO_4_ + ZnSO_4_).

The convergence of these three observations strongly supports preferential retention of Mg/Zn/S-bearing species in surface or near-surface environments, rather than extensive incorporation into the bulk calcite lattice. This conclusion in turn supports the Cabrera–Vermilyea framework of facet-selective step pinning as the operative mechanism. Mg^2+^ adsorbs preferentially at the acute step edges of the {104} face, where geometric and electrostatic constraints favor binding of the smaller, more strongly hydrated cation [10,11,35]. SO_4^2−^_ acts cooperatively, perturbing interfacial water structure and modifying the local kinetics of cation dehydration [14,15]. Concurrent Zn^2+^ dosing maintains the facet-selective surface population as the crystal grows beyond the initial Mg/SO_4_-poisoned shell [36,37]. The macroscopic outcome—biased growth toward elongation along directions less constrained by acute-step advancement, yielding the whisker-like crystalline nanorods habit observed by SEM and TEM (Figure 2c and Figure 3c)—is precisely what the facet-selective step-pinning scenario predicts.

It bears emphasis that this triangulated framework is not merely confirmatory but mechanistically discriminating. Each line of evidence rules out a different alternative explanation. The XRD result argues against substantial bulk lattice substitution within the detection limit of the present measurements. The multi-stage TGA signature rules out amorphous co-precipitate retention, which would produce a single broad thermal event. The EDS signal rules out trivial impurity surface contamination, which would not appear selectively in only one of the three products. Together, the three observations position the surface-adsorption mechanism not as one hypothesis among several, but as the explanation most consistent with the entire dataset.

Nonetheless, an important limitation of the present evidence should be acknowledged explicitly. XRD, TG/DTG, and EDS each probe the consequences of surface retention—lattice parameters, thermal decomposition behavior, and bulk-averaged elemental composition—rather than the chemical environment or spatial distribution of the Mg/Zn/S species themselves, and the surface-localization conclusion therefore rests on the convergence of indirect observations rather than on direct spectroscopic demonstration. Surface-sensitive techniques would provide the direct probe that the present dataset lacks: X-ray photoelectron spectroscopy (XPS) could quantify the near-surface Mg 2p, Zn 2p, and S 2p chemical states and their enrichment relative to the bulk; time-of-flight secondary ion mass spectrometry (ToF-SIMS) depth profiling could resolve the surface-to-bulk concentration gradient of each species; and STEM-EDS elemental mapping of individual whisker subunits could establish whether the additive-derived species decorate specific crystal facets, as the step-pinning mechanism predicts. Beyond the question of where the additive-derived species reside, direct verification of the step-pinning mechanism itself—that is, of how these species modify growth—would require time-resolved observation of step propagation on calcite surfaces in the presence of Mg^2+^, Zn^2+^, and SO_4^2−^_, for example, by in situ atomic force microscopy of the (104) face under controlled supersaturation, as has been performed for other calcite growth modifiers. The present study establishes the morphological outcome, the compositional fingerprint, and their internal consistency with facet-selective step pinning, while the elementary kinetic events underlying the mechanism remain to be resolved. These analyses were beyond the scope of the present study but are planned as follow-up work. Within the current dataset, the triangulated XRD/TGA/EDS framework establishes surface-dominant retention as the interpretation most consistent with all observations, while direct facet-resolved confirmation of the adsorption geometry awaits surface-sensitive characterization.

### 4.3. Crystallization by Particle Attachment in the Spindle/Rosette Route: Microstructural Visualization of Non-Classical Growth

The spindle route differs mechanistically from the whisker route in a fundamental way: the macroscopic habit emerges not from facet-selective ion-by-ion growth but from additive-directed assembly of pre-formed primary subunits—a pathway encompassed by the umbrella term crystallization by particle attachment (CPA) [16,17,21]. CPA pathways have been extensively characterized in biomimetic, polymer-templated, and confined-volume systems. Their explicit visualization on a gas–liquid carbonation platform operable at an industrially relevant scale, however, has remained rare. The TEM image of an isolated spindle particle reported here (Figure 3b) provides exactly that.

The spindle particle displays pronounced internal contrast striations running parallel to its long axis. Three considerations identify these striations as a microstructural signature of oriented attachment rather than an artifact of imaging or sample preparation. First, the striations are visible only along the long axis, with no analogous features perpendicular to it; this directional anisotropy excludes random bend contours or thickness fringes, neither of which would show a systematic preferred orientation in a fully crystallized single particle. Second, the striation spacing is consistent with the dimensions of primary subunits expected from CPA-mediated assembly of nanoscale precursors, not with the lattice fringes of a single calcite crystal—lattice fringes would require atomic-resolution imaging to resolve. Third, whisker-like crystalline nanorods of comparable size in the same TEM dataset (e.g., Figure 3c) show no comparable internal striations, indicating that the feature is specific to the spindle morphology rather than an artifact of the imaging conditions.

We interpret the spindle subunits as products of a two-stage formation sequence operating within the second carbonation stage of the route. In the first sub-stage, the NH_4_Cl-modified secondary slurry supplies a population of small anisotropic primary crystallites that nucleate on the pre-formed seeds and undergo limited classical growth, biased toward the *c*-axis by NH_4^+^_-directed adsorption at the carbonate-perpendicular surface terminations [35,38,39]. In the second sub-stage, these anisotropic primary crystallites undergo oriented attachment along matching crystallographic faces, generating the larger spindle architecture observed by TEM and SEM. The macroscopic rosette-like superstructures resolved at lower magnification in the same product (compare Figure 2b and Figure 3b at different magnifications) represent the next hierarchical level of assembly, in which spindle subunits themselves co-attach radially from a common nucleation point [16,40]. The decoupling of nucleation (Stage 1) from growth/assembly (Stage 2) introduced by the two-step protocol is essential to this hierarchical sequence, because it ensures that nucleation events do not compete with assembly events during Stage 2 [8].

The relevance of this observation extends beyond the specific spindle product. Direct microstructural evidence for CPA in calcite produced under industrially scalable conditions has been remarkably scarce in the published literature; most reports of oriented-attachment mechanisms in calcite originate from biomimetic or polymer-confined systems whose scalability is intrinsically limited [21,22,23]. The visualization reported here connects the well-established CPA framework to a process geometry—gas–liquid carbonation of Ca(OH)_2_ slurry—that is industrially mature and directly compatible with carbon-mineralization applications.

### 4.4. Implications: A Unified Design Framework and Its Industrial Significance

The mechanistic interpretations of Section 4.1, Section 4.2 and Section 4.3 collectively point to a design framework in which calcite habit on the gas–liquid carbonation platform is governed by two largely independent axes: (i) the degree of facet selectivity imposed by the additive system and (ii) the kinetic separation between nucleation and growth/assembly events imposed by the seeding strategy. The three habit routes reported here populate distinct regions of this two-dimensional design space. The Rn route operates with low facet selectivity and no kinetic separation, yielding the near-equilibrium equant habit. The whisker route operates with high facet selectivity but no kinetic separation, yielding kinetically biased 1D anisotropy. The spindle route combines moderate facet selectivity with explicit kinetic separation, yielding hierarchical assembly via CPA. Adjacent positions in this design space should correspond to morphological outcomes that share more features than they differ in, providing a predictive framework for accessing further habits—plate-like, dendritic, and hollow—within the same operational envelope.

Several implications follow that bear on practical deployment. First, the three routes can in principle be operated within the same physical reactor through recipe-level rather than equipment-level changes, a distinction that matters substantially for industrial capital expenditure. Morphology engineering thus becomes a high-value differentiator of carbon-mineralization processes without requiring dedicated reactor systems for each habit. Second, the value of morphology engineering is amplified by the steep gradient between bulk and morphology-refined carbonate prices (USD 30–80 vs. USD 300–1000+ per tonne). Even partial penetration of the morphology-refined market substantially improves project economics relative to carbon-mineralization processes that target only the carbon-sequestration value stream [2,5,41]. Third, the platform is compatible with industrial CaO-bearing residue streams—steel slag, lime sludge, and carbide slag [5,42,43]—linking the morphology engineering reported here to a broader circular economy context in which alkaline waste valorization, carbon sequestration, and value-added materials production are jointly delivered by a single process.

The study also illustrates the value of multi-technique triangulation in mechanism elucidation for habit-controlled crystallization. The surface-localization conclusion for the whisker route would have been substantially less defensible on any one of the three lines of evidence alone (XRD, TGA, or EDS); reviewer scrutiny of single-technique mechanism claims in the literature has, with good reason, become increasingly rigorous [16,44]. The cross-corroborative framework demonstrated here may serve as a methodological template for future habit-modification studies in carbonate and related mineral systems.

**Figure 7:**
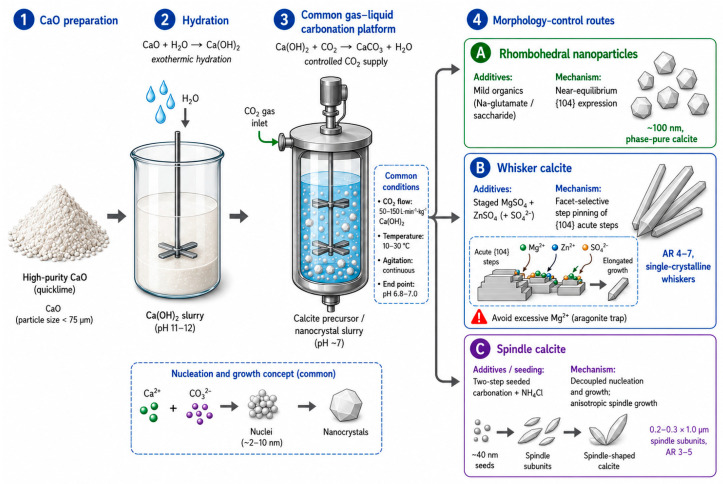
Schematic of the unified gas–liquid carbonation platform. The reactor, slaking chemistry, gas–liquid mass transfer, and operating envelope (10–30 °C; CO_2_ 50–150 L·min^−1^·kg^−1^ Ca(OH)_2_; pH 6.8–7.0) are held constant; only additive chemistry and seeding differ. Route I: sodium glutamate (pre-CO_2_) → Rn. Route II: staged MgSO_4_ (pre-CO_2_) + ZnSO_4_ (concurrent) → whiskers. Route III: two-step seeded carbonation with NH_4_Cl → spindles/rosettes.

Looking beyond the three habits explicitly demonstrated, the same platform should support a substantially broader design space along three further axes that are orthogonal to the additive-and-seeding levers explored here. First, primary-particle size can be tuned across the sub-50 nm to several-micrometer range by systematic adjustment of supersaturation history (CO_2_ partial pressure and flow rate, slurry temperature, and slaking dilution) and seeding density, variables that act primarily on the balance between nucleation and growth without disturbing the operating envelope. Second, the morphological repertoire should extend well beyond rhombohedral, whisker, and spindle habits into plate-like, dendritic, hollow, and porous architectures, accessible by combining additive families with complementary facet-binding selectivities—long-chain carboxylates, amino acid families, polyelectrolytes, and multi-cation cocktails—and by varying the staging sequence of additive introduction relative to CO_2_ injection. Third, controlled bulk chemical substitution—Mg-, Sr-, Mn-, and rare-earth-substituted calcites of tunable stoichiometry—is anticipated through partial or full replacement of CaO with mixed-cation alkaline precursors and through carbonation kinetics deliberately tuned to drive dopant uptake into the bulk lattice rather than retention at the surface, essentially inverting the surface-localization regime exploited here for the whisker route. Together, these three axes—size, morphological diversity, and bulk chemistry—define a design space substantially larger than the one populated by the present study, and the framework established here (calcite habit parameterized by additive facet selectivity and kinetic nucleation–growth decoupling) provides the conceptual scaffolding on which this broader space can be systematically explored. All three extensions are anticipated to be achievable within the same physical reactor through recipe-level rather than equipment-level changes, preserving the industrial scalability that distinguishes the platform from polymer-templated, membrane-confined, or biomimetic alternatives. This design framework is summarized in Figure 7.

### 4.5. Scale-Up Considerations: From Laboratory Mechanism to 20 kg Slurry Operation

A distinguishing feature of the present study is that all three habit-controlled products were synthesized at the 20 kg slurry scale rather than at the gram-to-tens-of-grams scale typical of laboratory calcite morphology studies. Operation at this scale introduces physical constraints that are largely absent from laboratory syntheses, and the behavior of the habit-control levers under these constraints warrants explicit discussion.

The first constraint is gas–liquid mass transfer. In small-volume laboratory systems, CO_2_ delivery can be maintained close to reaction-limited conditions so that the supersaturation experienced by the growing crystals reflects the intrinsic carbonation kinetics. In a 20 kg mechanically agitated slurry, by contrast, CO_2_ dissolution becomes transport-limited: the volumetric mass-transfer coefficient of the bottom-mounted gas ejector, rather than the intrinsic Ca(OH)_2_–CO_2_ reaction rate, sets the local supersaturation field. The habit-control strategies employed here are attractive precisely because they operate at the crystal–solution interface—facet-selective step pinning in Route II and seed-mediated growth in Route III act on step propagation and nucleation site availability, respectively—and therefore remain effective even when the supersaturation is delivered under transport-limited conditions. The phase purity and morphological uniformity of all three products (Section 3.2 and Section 3.3) demonstrate that this interfacial mode of control is robust to the heterogeneous supersaturation environment of the 20 kg reactor.

The second constraint is macroscopic mixing. A 20 kg slurry sustains spatial gradients in pH, dissolved CO_2_, and additive concentration on timescales comparable to or longer than local nucleation events. Two protocol-level design choices mitigate the influence of these gradients on habit selection. In Route II, MgSO_4_ is fully dispersed in the slurry before CO_2_ injection, ensuring that the step-pinning cation population is spatially uniform before the first nucleation events occur, while the concurrent ZnSO_4_ feed maintains the active surface population during growth. In Route III, nucleation and habit-selective growth are separated into two distinct carbonation stages so that neither process must compete with the other within a spatially heterogeneous environment. The recipe-level architecture of the platform is thus not merely operationally convenient but mechanistically matched to the mixing limitations of scaled operation.

The third constraint is thermal. Unlike laboratory syntheses with active thermostatting, the 20 kg batches undergo a spontaneous exotherm-driven temperature excursion (approximately 14-30 °C over ~6 h in Route II) arising from the carbonation enthalpy (ΔH°_rxn_ ≈ −113 kJ mol^−1^) under a decreasing surface-to-volume ratio. The operating envelope was deliberately positioned so that this uncontrolled excursion remains below the regime in which aragonite formation becomes kinetically competitive in Mg^2+^-bearing systems (Section 4.1); the phase-pure calcite obtained in all batches confirms that passive thermal management suffices at the 20 kg scale. At substantially larger scales, however, the surface-to-volume ratio decreases further and heat removal becomes progressively more demanding, so that active cooling or staged CO_2_ injection may be required to keep the temperature trajectory within the calcite-selective window.

These considerations also define the expected limitations of further scale-up beyond 20 kg. Volumetric mass-transfer coefficients and CO_2_ utilization efficiency generally decline with increasing vessel size unless gas dispersion is intensified, favoring multi-point or distributed gas injection in larger reactors. Macro-mixing times lengthen relative to nucleation timescales, placing a premium on the pre-dispersion and staged-dosing strategies already embedded in Routes II and III. Additive consumption, negligible at the 20 kg scale, becomes a cost factor at the tonne scale, although the low additive loadings used here (≤0.03 mol MgSO_4_ and 0.01–0.20 mol NH_4_Cl per mol Ca(OH)_2_) and the potential for recycling the Stage 1 seed suspension in Route III limit this burden. None of these constraints alters the underlying habit-control logic; rather, they define the engineering boundary conditions within which the two recipe-level levers must be deployed as the platform is scaled toward industrial carbon-mineralization applications.

## 5. Conclusions

We have demonstrated that three crystallographically equivalent but morphologically distinct calcite nanostructures—rhombohedral nanoparticles, whisker-like nanorods, and spindle/rosette assemblies—can be obtained from a unified CaO/Ca(OH)_2_-based gas–liquid carbonation platform. The three habits are accessed by changing only the additive chemistry and the seeding strategy; reactor configuration, slaking chemistry, and pH endpoint were maintained, whereas gas composition/flow and additive–seeding recipes were route-specific. The unified platform thus delivers morphological diversity through recipe-level rather than equipment-level differentiation.

Three mechanistic conclusions emerge from the multi-technique characterization. First, three independent lines of evidence—crystallographic (XRD), thermal (TGA/DTG), and chemical (EDS)—converge on a single conclusion for the whisker route: the Mg^2+^, Zn^2+^, and SO_4_^2−^ habit-modifying species are retained at the crystal surfaces of the nanoscale primary subunits rather than within the bulk calcite lattice. This convergence supports the Cabrera–Vermilyea framework of facet-selective step pinning as the operative mechanism and offers a methodological template for surface-versus-bulk discrimination in habit-modified carbonate systems. Second, TEM of an isolated spindle particle resolves internal contrast striations parallel to the long axis, which are more consistent with oriented attachment of pre-formed anisotropic primary subunits than with simple single-crystal growth—a non-classical pathway that has rarely been visualized directly on a scalable gas–liquid carbonation platform. Third, the morphological progression across the three routes can be rationalized within a two-axis design framework in which calcite habit on the carbonation platform is governed by the degree of facet selectivity imposed by the additive system and by the kinetic separation between nucleation and growth/assembly imposed by the seeding strategy. This framework provides predictive guidance for accessing further habits within the same operational envelope.

The unified platform established here clarifies the molecular-scale levers available to the practitioner for calcite habit engineering and positions morphology-engineered calcite as a value-added co-product of carbon-mineralization processes. By converting gaseous CO_2_ and CaO-bearing alkaline feedstocks into phase-pure crystalline products whose habit—and thus commercial value—is set by tunable solution chemistry alone, the platform links fundamental crystal-engineering science to the broader goals of carbon mineralization, industrial-residue valorization, and high-value materials manufacture from low-value precursors.

## Data Availability

The original contributions presented in this study are included in the article. Further inquiries can be directed to the corresponding author.
